# Anticoagulation-Associated Bleeding in Patients Screened for Atrial Fibrillation versus Usual Care—A Post Hoc Analysis from the LOOP Study

**DOI:** 10.1055/a-2202-4296

**Published:** 2024-01-08

**Authors:** Emilie Katrine Kongebro, Søren Zöga Diederichsen, Lucas Yixi Xing, Ketil Jørgen Haugan, Claus Graff, Søren Højberg, Morten S. Olesen, Derk Krieger, Axel Brandes, Lars Køber, Jesper Hastrup Svendsen

**Affiliations:** 1 Department of Cardiology, Copenhagen University Hospital—Rigshospitalet, Copenhagen, Denmark; 2 Department of Cardiology, Zealand University Hospital Roskilde, Roskilde, Denmark; 3 Department of Health Science and Technology, Aalborg University, Aalborg, Denmark; 4 Department of Cardiology, Bispebjerg Hospital, Copenhagen University Hospital, Copenhagen, Denmark; 5 Department of Biomedical Sciences, University of Copenhagen, Copenhagen, Denmark; 6 Mohammed Bin Rashid University of Medicine and Health Sciences, Dubai Healthcare City, Dubai, United Arab Emirates; 7 Department of Neurology, Mediclinic Parkview Hospital, Al Barsha South, Dubai, United Arab Emirates; 8 Department of Cardiology, Odense University Hospital, Odense, Denmark; 9 Department of Clinical Research, Faculty of Health Sciences, University of Southern Denmark, Odense C, Denmark; 10 Department of Cardiology, University Hospital of Southern Denmark—Esbjerg, Esbjerg, Denmark; 11 Department of Clinical Medicine, Faculty of Health and Medical Sciences, University of Copenhagen, Copenhagen, Denmark

**Keywords:** major bleeding, subclinical atrial fibrillation, implantable loop recorder screening, oral anticoagulation

## Abstract

**Background** Atrial fibrillation (AF) prevalence is rising; however, data on the bleeding risks associated with the detection of subclinical AF are needed.

**Objective** Our objective was to determine the bleeding increment associated with implantable loop recorder (ILR) screening for subclinical AF and subsequent anticoagulation initiation compared with usual care.

**Methods** This post hoc study utilized LOOP trial data from 6,004 elderly patients with stroke risks randomized to either ILR ( *n* = 1,503) or usual care ( *n* = 4,503). The mean follow-up time was 64.5 months, and none were lost to follow-up. The primary exposure was the initiation of oral anticoagulation, and the main outcome was the risk of major bleeding events following initiation of oral anticoagulants (OACs), determined by time-dependent cox regression. Second, we investigated antithrombotic prescription patterns and major bleeding events after antiplatelet treatment and in subgroups.

**Results** OAC was initiated in 1,019 participants with a mean age (years) of 78.8 (± 4.67) in control versus 77.0 (± 4.84) in ILR, *p* < 0.0001. Altogether did 202 participants end or pause OAC treatment. Among AF patients (n = 910) had 40 (28%) completely ended OAC and 105 (72%) temporarily paused OAC during follow-up. Major bleeding events totaled 221 (3.7%). Forty-seven major bleeding events followed an OAC initiation in 1,019 participants (4.6%); 26 versus 21 events in the control and ILR groups, respectively. The hazard ratio (HR) for major bleeding after OAC initiation compared with before initiation was 2.08 (1.50–2.90) *p* < 0.0001 overall, 2.81 (1.82–4.34) *p* < 0.0001 for control and 1.32 (0.78–2.23) *p* = 0.31 for the ILR group ( *p* = 0.07 for interaction). Antiplatelet treatment resulted in an overall adjusted HR of 1.3 (0.96–1.75) *p* = 0.09. For OAC users aged ≥75 years in the ILR group, the rate of major bleeding was 1.73 (0.92–2.96) compared with 0.84 (0.36–1.66) for an age <75 years, and the rate of the corresponding control subgroup aged ≥75 years was 2.20 (1.23–3.63) compared with 1.64 (0.82–2.93) for an age <75 years.

**Conclusion** The individual risk of major bleeding increased twofold after initiation of oral anticoagulation for all patients in this study. However, the patients screened for subclinical AF did not have a higher bleeding risk after initiation of anticoagulation compared with those in usual care.

**Trial Registration:** The LOOP study is registered at ClinicalTrials.gov, identifier: NCT020364 50

## Graphical abstract

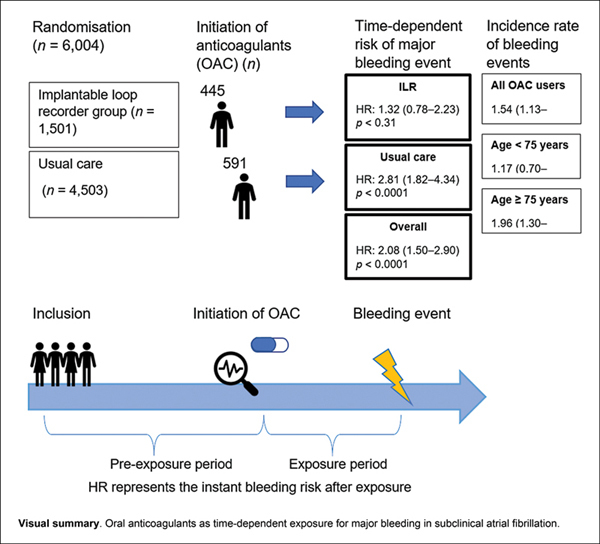

## Introduction

Oral anticoagulant (OAC) therapy is a cornerstone in the treatment of atrial fibrillation (AF) with the aim of preventing stroke. However, patients receiving OAC are at an increased risk of bleeding, which constitutes a serious nonnegligible cause of morbidity and mortality, and this risk increases with age. 1 2 Krijthe et al estimated a twofold increase in European AF prevalence from 2010 to 2060, 3 which may in turn increase the incidence of bleeding complications due to OAC. 4 5 These prevalence projections might even be underestimated in light of the recent years' accessibility to health monitoring, increasing burden of comorbidity and public awareness of AF. The LOOP study set out to prevent stroke by early detection of AF using implantable loop recorder (ILR) screening and subsequent early OAC initiation. 6 The study did not find a significantly reduced risk of stroke and therefore questioned whether all cases of AF are clinically relevant and inherently call for anticoagulant therapy. 6 The recent NOAH-AFNET 6 (non-vitamin K antagonist oral anticoagulants in patients with atrial high rate episodes) trial investigated this knowledge gap and found that not all subclinical AF should be treated with OAC. In this current post hoc analysis, we utilized LOOP study data to examine major bleeding events with OAC and antiplatelets as time-sensitive exposures, aiming to further investigate the downstream effects of screening for subclinical AF.

## Methods

### Study Design

The LOOP study was an open, randomized, controlled multicenter trial conducted in Denmark. The trial sought to prevent stroke by early detection and treatment of AF using ILR screening compared with usual care. The included participants were ≥70 years of age and diagnosed with at least one known risk factor for stroke: hypertension, diabetes, previous stroke, or heart failure. Exclusion criteria included pre-existing AF, treatment with heparin or OAC, and any contraindications to OAC. 7 Recruitment was conducted via the identification of potential participants using the Danish health registries followed by the letter of invitation. Upon initial visit at the study facility, eligibility was confirmed by obtaining a detailed medical history, concomitant medication, physical examination, a 12-lead electrocardiogram (ECG) to rule out AF, vital signs consisting of blood pressure (BP) measurement, heart rate, height and weight, as well as blood samples. Eligible participants were randomized in a one-to-three ratio to either continuous monitoring with an ILR (ILR group) or usual care (control group). All participants included in this trial gave informed consent before enrolment. Shortly after randomization, participants in the ILR group underwent ILR implantation allowing continuous ECG monitoring until death, withdrawal, or end of life of the device. AF episodes were adjudicated, and OAC treatment was initiated according to guidelines. 4 Besides annual on-site visits during the first 3 years for the ILR group and after 3 years for the control group, continuous ECG monitoring, data were also collected on an annual basis via remote contacts and look-up in medical records with the focus on hospital admissions, drug prescriptions, and outpatient visits. 7 The local Ethics Committee and the Danish Data Protection Agency approved the study (H-4-2013-025). The study was registered at ClinicalTrials.gov (NCT02036450).

### Outcomes

The primary outcome for this post hoc analysis was adjudicated major bleeding. We used the International Society on Thrombosis and Haemostasis definitions for major bleeding. 8 Events of major bleeding were registered by remote contact and/or look up in medical records annually or at the final assessment. Other outcomes included OAC initiation, OAC discontinuation, antiplatelet initiation, and antiplatelet discontinuation. All deaths were registered.

### Statistics

**Fig. 1:**
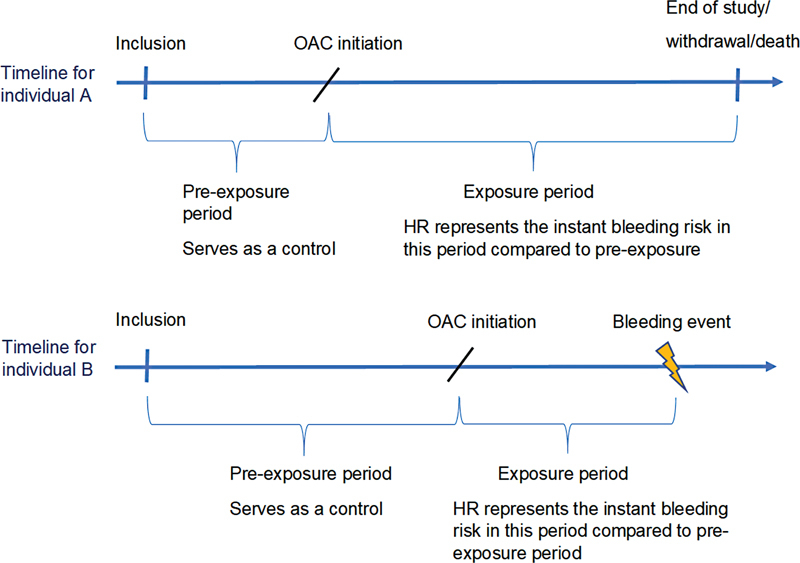
Example to illustrate the time-dependent analysis.

Baseline characteristics were presented as frequency with percent for categorical variables (compared by chi-squared tests), mean values ± standard deviation for continuous variables with normal distribution (compared by *t* -tests), or medians with first and third quartiles for continuous variables with nonnormal distributions (compared by Wilcoxon rank-sum tests). Incidence rates were all analyzed as events per 100 person-years. Cumulative incidences were estimated using the Aalen–Johansen estimator to account for death as competing risk. Incidence rates, cumulative incidences, and hazard ratios (HR) were presented with 95% confidence intervals (CI). Baseline variables analyzed as risk factors for major bleeding were age, hypertension, creatinine, previous stroke, previous transient ischemic attack (TIA), and alcohol consumption. This was done by time-to-event analysis both overall and within each randomization group. Time-dependent analyses were performed for the exposures of OAC and antiplatelets separately, to evaluate these as time-sensitive exposures to major bleeding in each individual. 14 Here, participants who received OAC during follow-up were analyzed as unexposed with time from baseline until censoring at the start of the treatment and as exposed hereafter ( Fig. 1 ), and similarly for antiplatelets albeit this analysis used time from initiation of antiplatelets or time from baseline if treated with antiplatelets already as the exposure. Participants with concurrent use of antiplatelets and OAC were not excluded from either analysis. The Cox proportional-hazards model was then applied to assess the relative risk of major bleeding regarding exposure initiation. A sensitivity analysis censoring baseline antiplatelets was performed to only assess incident antiplatelets. Supplementary models included multivariate adjustment for sex, age, baseline plasma creatinine, previous stroke (as specified in the main publication 6 ), TIA, systemic arterial embolism (SAE), systolic blood pressure (SBP), and alcohol consumption. Subgroup analyses were performed across randomization groups and treatment with OAC, antiplatelets or none of these in addition to age (<75 and ≥75 years), sex, baseline plasma creatinine (<100 and ≥100), and SBP (<150 and ≥150 mm Hg) with tests for interaction between these exposures and randomization group. Assumptions for Cox proportional hazards modeling tested with Schoenfeld and Martingale residuals were appropriate, and any violations were reported. *p* -Values < 0.05 were considered statistically significant.

## Results

Between January 2014 and May 2016, a total of 6,004 participants were enrolled in the trial. Table 1 displays the baseline characteristics in the study population. Overall, 47% were women, the mean age was approximately 75 years, 91% were diagnosed with hypertension, 48% received antiplatelets at baseline, and none were prescribed with anticoagulation. Last follow-up data were obtained in January 2021 with a median follow-up time of 64.5 months [59.3,69.8]. None were lost to follow-up. The LOOP study population has been described thoroughly in previous publications. 6 9

**Table 1:** Baseline characteristics

	Control ( *n* = 4,503)	ILR ( *n* = 1,501)	Total ( *n* = 6,004)
Age, years	74.7 ± 4.1	74.7 ± 4.1	74.7 ± 4.1
Male sex	2,375 (52.7)	792 (52.8)	3,167 (52.7)
Smoking status			
Previous	2,302 (51.1)	769 (51.2)	3,071 (51.2)
Current	417 (9.3)	135 (9.0)	552 (9.2)
Never	1,782 (39.6)	597 (39.8)	2,379 (39.6)
Alcohol consumption, units per week	5 (1–10)	5 (1–10)	5 (1–10)
Diagnoses			
Hypertension	4,066 (90.3)	1,378 (91.8)	5,444 (90.7)
Previous AMI or CABG	494 (11.0)	130 (8.7)	624 (10.4)
Diabetes	1,288 (28.6)	422 (28.1)	1,710 (28.5)
Previous stroke, transient ischemic attack or systemic arterial embolism	1,139 (25.3)	370 (24.7)	1,509 (25.1)
Peripheral arterial disease	392 (6.5)	88 (5.9)	304 (6.8)
Valvular heart disease	181 (4.0)	63 (4.2)	244 (4.1)
Heart failure	199 (4.4)	67 (4.5)	266 (4.4)
CHADS-VASc	4 (3–4)	4 (3–4)	4 (3–4)
2	588 (13.1)	202 (13.5)	790 (13.2)
3	1,494 (33.2)	513 (34.2)	2,007(33.4)
4	1,325 (29.4)	419 (27.9)	1,744 (29.0)
5	687 (15.3)	244 (16.3	931(15.5)
≥6	409 (9.1)	123 (8.2)	532 (8.9)
Physical			
Systolic BP, mm Hg	149.8 ± 19.5	150.6 ± 19.2	150 ± 19.4
Diastolic BP, mm Hg	83.9 ± 11.3	84.7 ± 11.1	84.1 ± 11.2
Pulse, beats per minute	71.3 ± 12.5	71.6 ± 12.1	71.4 ± 12.4
BMI, kg/m ^2^	27.6 ± 4.5	27.8 ± 4.7	27.7 ± 4.6
Creatinine, μmol/L	85.8 ± 26.2	84.8 ± 24.2	85.6 ± 25.7
Prescriptions			
Antiplatelets	2,204 (48.9)	702 (46.8)	2,906 (48.4)
Beta-blockers	1,172 (26.0)	354 (23.6)	1,526 (25.4)
Diuretics	1,511 (33.6)	495 (33.0)	2,006 (33.4)
Statins	2,621 (58.2)	879 (58.6)	3,500 (58.3)
Renin-angiotensin inhibitors	2,999 (66.6)	991 (66.0)	3,990 (66.5)
Ca-blockers	1,684 (37.4)	562 (37.4)	2,246 (37.4)
Insulins	354 (7.9)	124 (8.3)	478 (8.0)
Other antidiabetics	959 (21.3)	328 (21.9)	1,287 (21.4)

Abbreviations: AMI, acute myocardial infarction; BMI, body mass index; BP, blood pressure; CABG, coronary artery bypass graft; ILR, implantable loop recorder; SAE, systemic arterial embolism; TIA, transient ischemic attack.

### Antithrombotic Prescriptions

**Fig 2:**
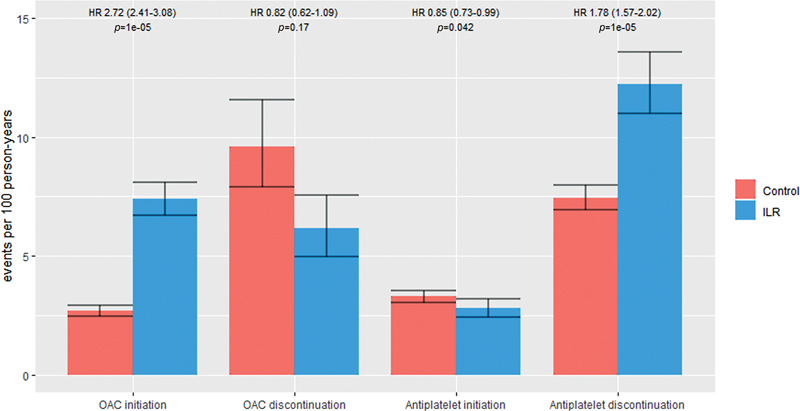
Changes in antithrombotic treatments during the study period. Event rates and hazard ratios for OAC and antiplatelet initiation and discontinuation calculated as events per 100 person-years since drug-naive baseline or start of the respective drug in the control and ILR group. Error bars indicate 95% confidence interval.

Changes in prescriptions for OAC and antiplatelets were registered during follow-up ( Table 2 and Fig. 2 ). OAC was initiated by 1,036 (17%) participants for any indication, and the ILR group had a significantly higher initiation rate compared with control (HR: 2.72, 95% CI: 2.41–3.08, *p* < 0.0001) and were approximately 2 years younger at initiation (77.0 ± 4.84 years) compared with control group (78.8 ± 4.67 years [ *p* < 0.0001]). All-cause OAC discontinuation occurred in 202 participants (19%) during follow-up and of these 118 participants reinitiated again their OAC treatment for any indication within a median of 0 days (interquartile range (IQR) = 37). The reinitiation of OAC was more common in the ILR group (68%) compared with the control group (49%). Specifically, for AF-diagnosed participants who initiated OAC ( *n* = 910), a total of 145 paused OAC treatment, and of these 62 participants (43%) switched quickly to a different OAC or dosage within 24 hours and 43 participants (29%) reinitiated later in follow-up. In total, 40 participants (28%) completely ended OAC treatment at the end of follow-up. Complete discontinuation ( *n* = 84) was more common for AF patients in the ILR group ( *n* = 24 in ILR vs. *n* = 16 in control) and much more common for non-AF-patients in the control group ( *n* = 41 in control vs. *n* = 3 in ILR group). Antiplatelet treatment was initiated in 905 (15%) participants during follow-up, with slightly more individuals in the control group. Overall, discontinuation of antiplatelets (baseline + incident) occurred in 1,107 cases with the highest rate in the ILR group.

**Table 2:** Changes in all OAC and antiplatelet prescriptions

Events	*n* (%)		Cumulative incidence at 5 y (95% CI) a		Incidence rate (95% CI) a		Unadjusted HR (95% CI), *p* -value b
	Control	ILR	Control	ILR	Control	ILR	
	*n* = 4,503	*n* = 1,501					
**OAC**							
I **nitiation**	591 (13.1)	445 (29.6)	11.65 (10.7–12.59)	29.53 (27.21–31.86)	2.68 (2.46–2.9)	7.38 (6.71–8.1)	2.72 (2.41–3.08), < 0.0001
A ** ll events of discontinuation c**	111 (18.8)	91 (20.4)	25.89 (20.97–30.82)	21.49 (17.47–25.5)	9.60 (7.9–11.56)	6.15 (4.95–7.55)	0.82 (0.62–1.09), 0.17
D **iscontinuation followed by reinitiation**	54 (48.6)	64 (70.3)					
C **omplete discontinaution: no AF**	41 (36.9)	3 (3.3)					
C **omplete discontinuation: with AF**	16 (14.4)	24 (26.4)					
**A** ntiplatelet							
I **ncident initiation**	706 (15.7)	199 (13.3)	14.3 (13.26–15.33)	12.17 (10.51–13.84)	3.29 (3.05–3.54)	2.79 (2.41–3.2)	0.85 (0.73–0.99), 0.042
D ** iscontinuation d**	758 (26.0)	349 (38.7)	27.32 (25.59–29.05)	43.07 (39.63–46.51)	7.44 (6.92–7.99)	12.2 (10.99–13.59)	1.78 (1.57–2.02), <0.0001

Abbreviations: AF, atrial fibrillation; CI, confidence interval; HR, hazard ratio; ILR, implantable loop recorder; OAC, oral anticoagulant.

a Calculated as events per 100 person-years.

b Comparing the control and ILR group.

c Calculated from OAC initiation until date of discontinuation.

d Calculated from baseline if initiated before baseline, or time of initiation if initiated during follow-up, until discontinuation.

### Incidence Rates for Major Bleeding According to Exposure

**Fig. 3:**
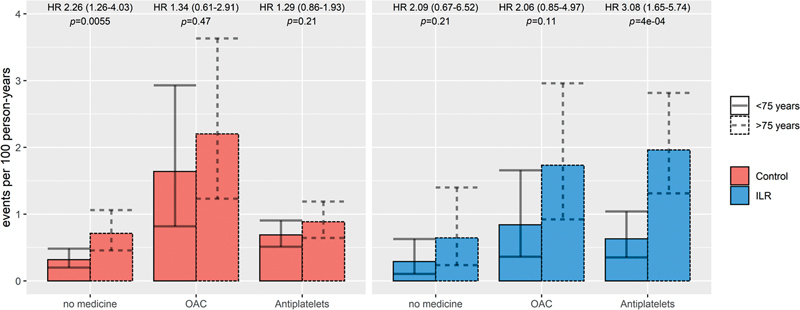
Major bleeding rates according to age and antithrombotic exposure in each randomization group. Event rates for major bleeding after initiation of drugs in the control and ILR groups compared for age groups <75 versus ≥75 years. Time to event is calculated as time from baseline or start of the respective drug to event or censoring, with censoring at drug initiation for those with no-treatment at baseline. Error bars indicate 95% confidence interval.

The incidence rates for major bleeding in the population subgroups are displayed in Table 3 . A total of 221 all-cause major bleedings occurred, with an overall cumulative incidence at 5 years of 3.99%.. (3.45–4.53), and an overall incidence rate of 0.72 (0.63–0.82) per 100 person-years with an HR of 1.26 (0.95–1.69), *p* = 0.11 for ILR versus control. A total of 2,201 participants were included in the “no-treatment” analysis, which resulted in a bleeding rate of 0.44 (0.33–0.57), with a significantly higher rate among participants aged ≥75 years compared with <75 years ( Fig. 3 ). An age ≥75 years were generally associated with increased bleeding rates, as seen to be significantly increased for the ILR group with antiplatelets as drug exposure. The performed interaction analyses were significant between the ≥75 age subgroup and for both OAC and antiplatelets, but not between the randomization groups and OAC or antiplatelets alone. For all AF patients, the cumulative incidence of major bleeding was 7.43% (5.75–9.11), with an incidence rate of 1.44 (1.13–1.8) and an HR of 0.92 (0.58–1.45), *p* = 0.72 for ILR versus control.

**Table 3:** Major bleeding rates by exposure group

Exposure group	Subgroups	Bleeding events, n (%)	Incidence rate (95% CI)	HR (95% CI), unadjusted	P for difference
**Without OAC or antiplatelets**					
**All**	All ( *n* = 2201) Control ( *n* = 1701) ILR ( *n* = 500) Age < 75 ( *n* = 1537) Age≥75 ( *n* = 664)	58 (2.6 %) 46 (2.7 %) 12 (2.4 %) 28 (1.8 %) 30 (4.5 %)	0.44 (0.33-0.57) 0.45 (0.33-0.60) 0.40 (0.21-0.70) 0.31 (0.21-0.45) 0.70 (0.47-1.00)	1 (reference) 0.89 (0.47-1.68) 1 (reference) 2.20 (1.33-3.73)	0.71 0.002
**ILR**	Age < 75 ( *n* = 359) Age≥75 ( *n* = 141)	6 (1.7 %) 6 (4.3 %)	0.29 (0.11-0.63) 0.64 (0.24-1.40)	1 (reference) 2.09 (0.67-6.52)	0.21
**CONTROL**	Age < 75 ( *n* = 1178) Age≥75 ( *n* = 523)	22 (1.9 %) 24 (4.5 %)	0.32 (0.20-0.48) 0.71 (0.46-1.06)	1 (reference) 2.26 (1.26-4.03)	0.006
**With OAC**					
**All**	All ( *n* = 1019) Control (n = 578) ILR (n = 441) Age < 75 ( *n* = 537) Age≥75 ( *n* = 482)	47 (4.6 %) 26 (4.5 %) 21 (4.8 %) 19 (3.5 %) 28 (5.8 %)	1.54 (1.13-2.05) 1.92 (1.26-2.82) 1.23 (0.76-1.89) 1.17 (0.70-1.83) 1.96 (1.30-2.83)	1 (reference) 0.68 (0.38-1.22) 1 (reference) 1.66 (0.93-2.98)	0.2 0.09
**ILR**	Age < 75 ( *n* = 242) Age≥75 ( *n* = 199)	8 (3.3 %) 13 (6.5 %)	0.84 (0.36-1.66) 1.73 (0.92-2.96)	1 (reference) 2.06 (0.86-4.97)	0.11
**CONTROL**	Age < 75 ( *n* = 295) Age≥75 ( *n* = 283)	11 (3.7 %) 15 (5.3 %)	1.64 (0.82-2.93) 2.20 (1.23-3.63)	1 (reference) 1.34 (0.61-2.91)	0.47
**With antiplatelets**					
**All**	All ( *n* = 3374) Control ( *n* = 2568) ILR ( *n* = 806) Age < 75 ( *n* = 2036) Age≥75 ( *n* = 1338)	139 (4.1 %) 95 (3.7 %) 44 (4.0 %) 66 (3.2 %) 73 (5.5 %)	0.86 (0.72-1.01) 0.77 (0.62-0.94) 1.14 (0.83-1.53) 0.67 (0.52-0.86) 1.13 (0.89-1.42)	1 (reference) 1.49 (1.04-2.13) 1 (reference) 1.68 (1.20-2.35)	0.03 0.002
**ILR**	Age < 75 ( *n* = 488) Age≥75 ( *n* = 318)	15 (3.1 %) 29 (9.1 %)	0.63 (0.35-1.04) 1.96 (1.31-2.82)	1 (reference) 3.08 (1.65-5.74)	0.0004
**CONTROL**	Age < 75 ( *n* = 1548) Age≥75 ( *n* = 1020)	51 (3.3 %) 44 (4.3 %)	0.69 (0.51-0.91) 0.89 (0.64-1.19)	1 (reference) 1.29 (0.86-1.93)	0.21

All participants were included in the analysis and could appear in several exposure groups, pending change in medical treatment. In each exposure group, rates were calculated as time from baseline or start of the treatment until event or censoring which included initiation of treatment for the “no treatment”-exposure group.

Abbreviations: ILR, implantable loop recorder; HR, hazard ratio; OAC, oral anticoagulants; CI, confidence interval

### Risk of Major Bleeding Using Time-Dependent Analysis

The unadjusted HR for major bleeding upon OAC initiation (including those with baseline antiplatelets) compared with before initiation was 2.37 (1.71–3.28), *p* < 0.0001 for the whole study population ( Table 4 ). The control group had a numerically higher risk compared with the ILR group, and the *p* -value for interaction between randomization groups and the exposure was 0.07. Multivariable adjustment confirmed these results, and furthermore found bleeding to be associated with higher age and prior stroke, TIA, or SAE. For antiplatelets (baseline + incident) as the exposure, the unadjusted HR for major bleeding was 1.52 (1.16–2.00), *p* = 0.0025, and this association was indifferent between the groups with a *p* -value for the interaction of 0.19. Multivariate adjustment yielded an HR of 1.30 (0.96–1.75), *p* = 0.086. A sensitivity analysis including only incident antiplatelets resulted in an overall HR of 1.14 (0.85–1.54), *p* = 0.38 ( Supplementary Table S3 [available in the online version]).

**Table 4:** Time-dependent analysis of bleeding risk upon drug initiation

Groups	Unadjusted HR (95% CI)	*p* -Value		Adjusted HR a (95% CI)	*p* -Value
**OAC**					
** All**	2.37 (1.71–3.28)	<0.0001		2.08 (1.50–2.90)	<0.0001
			Male sex	0.99 (0.73–1.35)	0.96
			Age, per year	1.09 (1.06–1.12)	<0.0001
			Creatinine, per 10 µmol/L	1.03 (0.98–1.08)	0.24
			Stroke or TIA or SAE	1.40 (1.03–1.90)	0.033
			Systolic BP, per 10 mm Hg	1.01 (0.95–1.09)	0.67
			Baseline antiplatelets	1.12 (0.83–1.51)	0.45
			Alcohol consumption, units per week	1.00 (0.98–1.02)	0.92
** Control**	3.19 (2.07–4.90)	<0.0001		2.81 (1.82–4.34)	<0.0001
** ILR**	1.63 (0.97–2.75)	0.065		1.32 (0.78–2.23)	0.31
**Antiplatelet**					
** All**	1.52 (1.16–2.00)	0.0025		1.30 (0.96–1.75)	0.086
			Male sex	0.99 (0.73–1.35)	0.97
			Age, per year	1.10 (1.07–1.13)	<0.0001
			Creatinine, per 10 µmol/L	1.03 (0.98–1.08)	0.23
			Stroke or TIA or SAE	1.34 (0.99–1.82)	0.06
			Systolic BP, per 10 mm Hg	1.02 (0.95–1.09)	0.66
			Alcohol consumption, units per week	1.00 (0.98–1.02)	0.80
** Control**	1.36 (0.99–1.88)	0.060		1.21 (0.85–1.72)	0.30
** ILR**	2.05 (1.22–3.46)	0.0066		1.56 (0.89–2.74)	0.12

Abbreviations: BP, blood pressure; CI, confidence interval; HR, hazard ratio; ILR, implantable loop recorder; OAC, oral anticoagulant; SAE, systemic arterial embolism; TIA, transient ischemic attack.

a Adjusted for sex, age, creatinine, previous stroke or TIA or SAE, SBP, alcohol consumption, and baseline antiplatelets.

## Discussion

This post hoc analysis used data from a randomized multicenter AF-screening trial including 6,004 older individuals, with stroke risk factors to investigate the risk of major bleeding following ILR-screening versus usual care (control group). We report three key findings. First, OAC initiation caused a twofold risk increase in every patient's personal bleeding risk compared with before initiation, independently of age, sex, and comorbidities. Second, antiplatelet treatment did increase the risk of bleeding compared with the period without treatment, but this finding was not robust to adjustment for confounders. Third, approximately one out of five participants either changed OAC type or discontinued OAC all together, and one out of three participants discontinued antiplatelet treatment.

### Antithrombotic Prescription Patterns

The ILR group had a higher OAC initiation rate, higher antiplatelet discontinuation rate, and a higher OAC reinitiation compared with the control group. Both randomization groups had high rates of OAC discontinuation, although highest in the control group together with a significantly increased rate of antiplatelet initiation. The observed prescription patterns in the study were much in line with our expectations as (1) OAC initiation was higher in the ILR group due to the increased AF detection rates in this group. (2) To initiate OAC in the ILR group, any current antiplatelets would usually have been discontinued. (3) The indications for OAC discontinuation were not registered in the trial. Reinitiation of OAC was common and with a high rate especially in the ILR group, which means that the overall discontinuation more often was temporary. Complete discontinuation of OAC could stem from intolerable side effects, major or minor bleeding events, or could be planned due to a temporary treatment regime from, e.g., venous thromboses. Furthermore, nonadherence should always be considered since this is common and does increase the risk of stroke and mortality in AF patients. 10 11 (4) It is noteworthy that in previous years, antiplatelets were largely prescribed even as a primary preventive measure to elderly individuals, which could have influenced the rates of both antiplatelet initiation and discontinuation in this study. 12 13 (5) The lower OAC initiation rate in the control group left more room to receive antiplatelets for newly diagnosed illnesses in the control group, as antithrombotic treatment must often yield when OAC is prescribed.

### Bleeding Rates

Older participants (≥75 years) had numerically increased bleeding rates in all treatment groups. The risk was significantly increased even without the use of antiplatelets or OAC in the control group and with the use of antiplatelets in the ILR group. Interaction analyses confirmed the different effects across all age and treatment subgroups. Older participants in the ILR group had an increased bleeding rate with antiplatelets; however, this was not robust to adjustments for hemorrhagic risk factors in the time-dependent analysis of antiplatelets in the ILR group. Furthermore, few participants were treated with OAC and antiplatelets in combination, which hamper the ability to further assess subgroups with increased bleeding risk following antiplatelets. Subgroup analyses on sex, baseline creatinine, and SBP did not reveal significant differences, although this has been reported as significant risk factors of major bleeding in OAC-treated AF patients. 1 5

### Time-Dependent Analysis on Bleeding Risk after Antithrombotic Exposure

Time-dependent analyses enable individuals to have his or her own control by estimating a risk before and after an exposure. The increase in bleeding risk was robust to adjustment for hemorrhagic risk factors, though this was not the case in both randomization groups: in the control group, the risk rose almost threefold, while the 30% increase in the ILR group did not reach statistical significance. The interaction between the exposure and randomization group was not significant but leaned toward increased OAC-mediated bleeding risk in the control group only, despite the rate of OAC initiation being three times higher in the ILR group. Several factors may influence the results: (1) the trial was initially powered to detect stroke and not major bleeding, (2) participants in the control group were approximately 2 years older at the time of OAC initiation, (3) the indication for OAC treatment differed between the groups; AF was the reason in 81% of the cases in the control group and 91% of the cases in the ILR group. This could indicate a further physical deterioration due to other illnesses and thereby an enhanced bleeding risk in the control group, and (4) AF in the control group was typically symptomatic AF, which could indicate a heavier cardiovascular burden compared with the diagnoses of asymptomatic, subclinical AF in the ILR group. However, treatment of device-detected subclinical AF could lead to a higher risk of bleeding but without the benefit of stroke prevention due to lower stroke risk associated with this arrhythmia as reported in the recently published NOAH-AFNET 6 trial. 15

The time-dependent analysis of antiplatelets did not reveal a significantly increased risk of bleeding in this study. This is in contrast to several studies reporting antiplatelet monotherapy or concurrent treatment with OAC to increase the risk of bleeding. 16 17 The lack of association could, besides too few events, be caused by healthy user bias (or other selection bias) as more risk-prone individuals may less often have accepted the study invitation, 18 the high antiplatelet discontinuation rate upon AF detection and initiation of OAC, or the per-protocol exclusion from enrolment of individuals with very high bleeding risk and contraindications to OAC treatment. 7

### Clinical Perspectives on Bleeding Risk

We report overall low rates of adjudicated major bleeding following OAC. An observational study by Van Rein et al reported a major bleeding rate of 2.3 per 100 person-years following OAC in more than 250,000 Danish AF patients during 4 years of follow-up. 2 We report a lower event rate for both OAC-treated patients overall and AF patients, despite a longer study follow-up, high rate of OAC initiation, and many participants treated relatively aggressively (at detection of low-burden AF). Furthermore, Van Rein et al reported a much higher bleeding rate in the nontreated group compared with the bleeding rates reported in this manuscript. This corroborates with real-world studies that often report higher occurrence of major bleeding, possibly due to the nontrial setting and nonadjudicated bleeding events along with possible nonadherence to clinical recommendations and a heavier comorbidity burden. 19 20 Our results confirm that older individuals in this population are particularly at risk of major bleeding besides the increased risk of thromboembolic events, as previous studies have reported in OAC-treated patients. 21 22 An American observational study used OAC as a time-dependent exposure in an older AF population and found an increased risk of major bleeding after OAC initiation (HR: 1.57; 95%CI: 1.54–1.59). 23 Our findings indicate a larger increase in OAC-mediated bleeding risk, despite a lower mean age and lighter comorbidity burden compared with the American cohort. The percentage of AF patients treated with OAC in the American cohort was low (41%) compared with the current study (87.1%), and yet the registry reported a significantly lower all-cause and inpatient medical cost for OAC-treated AF patients compared with their nontreated cohort. 23 With the expected surge in AF prevalence, 3 24 25 this underlines that a suited screening strategy is needed to identify and treat AF to mitigate mortality, morbidity, and health care expenses. 26 27 But before this, evidence is needed with regard to OAC treatment in subclinical AF. 15 Indeed, screening AF is gaining momentum but remains unknown territory with respect to side effects—that is, early detection and treatment may come with a different risk-benefit trade-off compared with what is known from conventionally diagnosed AF. 15 28 As a final perspective, the detection rate of low-burden AF is expected to rise further with the increasing use of consumer-operated devices and so-called wearables. 28

## Limitations

This was a post hoc analysis with its inherent limitations due to exploratory analyses and the risk of bias. Participants were all recruited from home by letter, which may inflict a healthy user bias in the recruitment. Patients with contraindications to OAC treatment were excluded from this trial, which may have introduced selection bias. Participants in a trial may have increased symptom awareness earlier than if not enrolled. The sample size might be insufficient to assess rare events such as major bleeding in a mostly un-anticoagulated population. Follow-up was limited to approximately 5.5 years, and follow-up for new exposures after baseline was inherently shorter, further limiting the statistical power to assess outcome. Nevertheless, the current study comprised a well-characterized randomized trial of a patient population not previously investigated for bleedings, namely anticoagulation of screen-detected subclinical AF versus usual care.

## Conclusion

All individuals who initiated OAC in this study experienced a doubling in personal bleeding risk compared with before initiation. However, individuals screened for subclinical AF did not have a higher bleeding risk after OAC initiation compared with those in usual care. Antiplatelets did not seem to increase the overall risk of bleeding in this study; however, older patients ≥ 75 years of age endured higher bleeding rates in all exposure groups in this study.

## Notes

**“What is Known about this Topic?”**

- Atrial fibrillation prevalence is projected to double within the next four decades, and the arrival of handheld screening technology may further add to the detection rates of asymptomatic atrial fibrillation.
- The harms and benefits of screening for subclinical atrial fibrillation are not well described.
- Novel data are needed to update screening guidelines for atrial fibrillation.

**“What does this Paper Add?”**

- We present trial data on the bleeding risk associated with screening for subclinical atrial fibrillation in elderly patients with stroke risks.
- Our main result displays a twofold risk increment of a major bleeding event following initiation of oral anticoagulants in all patients.
- However, the patients screened for subclinical atrial fibrillation did not have a higher bleeding risk compared with usual care.
