# Supplementary material for: Anticoagulation-Associated Bleeding in Patients Screened for Atrial Fibrillation versus Usual Care—A Post Hoc Analysis from the LOOP Study
Source: TH Open. 2024 Jan 8;8(1):e19–30. doi: 10.1055/a-2202-4296 (PMC10774012; doi:10.1055/a-2202-4296)
Supplement: Supplementary file 1 — Supplementary Material [file 10-1055-a-2202-4296-s23090038.pdf]

Supplementary Table S1 Baseline characteristics grouped by antiplatelet treatment at baseline

	Antiplatelets ($n = 2,906$)	No antiplatelets ($n = 3,098$)	p for difference
Age	75.0 ± 4.2	74.5 ± 4.0	<0.0001
Male sex	1,783 (61.4)	1,384 (44.7)	<0.0001
Control group	2,204 (75.8)	2,299 (74.2)	0.15
ILR group	702 (24.2)	799 (25.8)	0.15
Smoking status			<0.0001
Never	1,024 (35.2)	1,355 (43.7)	
Previous	1,569 (53.9)	1,502 (48.5)	
Current	312 (10.7)	240 (7.7)	
Hypertension	2,542 (87.5)	2,902 (93.7)	<0.0001
Alcohol consumption, units per week	7.4 ± 8.1	7.0 ± 8.1	0.06
Diabetes	894 (30.8)	816 (26.3)	0.0002
Previous stroke, transient ischemic attack, or systemic arterial embolism	1,250 (43.0)	259 (8.4)	<0.0001
Valvular heart disease	155 (5.3)	89 (2.9)	<0.0001
Previous AMI or CABG	522 (18.0)	102 (3.3)	<0.0001
Heart failure	204 (7.0)	204 (2.0)	<0.0001
CHA_2DS_2-VASc	4 (3,5)	3 (3,4)	<0.0001
2	230 (7.9)	560 (18.1)	
3	648 (22.3)	1359 (43.9)	
4	918 (31.6)	826 (26.7)	
5	683 (23.5)	248 (8.0)	
≥6	427 (14.7)	105 (3.3)	
Systolic BP, mmHg	148.4 ± 19.5	151.4 ± 19.2	<0.0001
Diastolic BP, mmHg	82.7 ± 11.1	85.5 ± 11.1	<0.0001
Pulse, beats per minute	70.1 ± 12.4	72.6 ± 12.3	<0.0001
BMI, kg/m^2	27.7 ± 4.4	27.7 ± 4.7	0.69
Creatinine, μmol/L	89.2 ± 27.4	82.1 ± 23.5	<0.0001
Medication			
Antiplatelets	2,906 (100.0)	0 (0.0)	<0.0001
Statins	2,218 (76.3)	1,282 (41.4)	<0.0001
Diuretics	956 (32.9)	1,050 (33.9)	0.43
Ca-blockers	1,117 (38.4)	1,129 (36.4)	0.12
B-blockers	945 (32.5)	581 (18.8)	<0.0001
Renin-angiotensin inhibitors	1,935 (66.6)	2,055 (78.4)	0.86
Insulins	263 (9.1)	215 (6.9)	0.003
Other antidiabetics	704 (24.2)	583 (18.8)	<0.0001

Abbreviations: AMI, acute myocardial infarction; BMI, body mass index; BP, blood pressure; CABG, coronary artery bypass surgery; ILR, implantable loop recorder.
Note: Missing values were creatinine 31, smoking status 2, alcohol consumption 3, systolic BP 7, diastolic BP 7, pulse rate 21, and BMI 1.

 © 2024. The Author(s).

Supplementary Table S2 Major bleeding events according to drug exposure grouped by randomization arm, creatinine, and blood pressure

Exposure groups	No. events (%)	Incidence rate (95% CI)	HR (unadjusted) (95% CI)	*p* Value	*p* interaction[a]
All (*n* = 6,004)	221 (3.7%)	0.72 (0.63–0.82)	1 (reference)		
No OAC or antiplatelets					
All (*n* = 2,201)	58 (1.9%)				
All—creatinine < 100 µM (*n* = 1,838)	45 (2.4%)	0.40 (0.29–0.54)	1		
All—creatinine ≥ 100 µM (*n* = 347)	13 (3.7%)	0.64 (0.34–1.09)	1.57 (0.85–2.91)	0.15	0.75
All—systolic B*p* < 150 (*n* = 1,085)	24 (2.2%)	0.37 (0.24–0.56)	1		
All—systolic BP ≥ 150 (*n* = 1,112)	34 (3.1%)	0.50 (0.34–0.70)	1.33 (0.79–2.24)	0.29	0.31
ILR—creatinine < 100 µM (*n* = 426)	7 (1.6%)	0.27 (0.11–0.56)	1		
ILR—creatinine≥100 µM (*n* = 70)	5 (7.1%)	1.20 (0.39–2.79)	4.4 (1.40–13.89)	0.011	
ILR—systolic B*p* < 150 (*n* = 227)	6 (2.6%)	0.45 (0.16–0.98)	1		
ILR—systolic BP≥150 (*n* = 272)	6 (2.2%)	0.36 (0.13–0.78)	0.79 (0.26–2.46)	0.69	
Control—creatinine < 100 µM (*n* = 1,412)	38 (2.7%)	0.44 (0.31–0.61)	1		
Control—creatinine ≥ 100 µM (*n* = 277)	8 (2.9%)	0.49 (0.21–0.97)	1.11 (0.52–2.37)	0.80	
Control—systolic B*p* < 150 (*n* = 858)	18 (2.1%)	0.35 (0.21–0.56)	1		
Control—systolic BP ≥ 150 (*n* = 840)	28 (3.3%)	0.54 (0.36–0.79)	1.53 (0.85–2.77)	0.16	
OAC					
All (*n* = 1,019)	47 (4.6%)				
All—creatinine < 100 µM (*n* = 773)	38 (4.9%)	1.64 (1.16–2.25)	1		
All—creatinine≥100 µM (*n* = 242)	9 (3.7%)	1.24 (0.57–2.36)	0.77 (0.37–1.58)	0.47	0.09
All—systolic BP < 150 (*n* = 488)	19 (3.9%)	1.31 (0.79–2.05)	1		
All—systolic BP ≥ 150 (*n* = 531)	28 (5.3%)	1.74 (1.16–2.52)	1.32 (0.74–2.36)	0.35	0.20
ILR—creatinine < 100 µM (*n* = 340)	17 (5.0%)	1.30 (0.76–2.08)	1		
ILR—creatinine ≥ 100 µM (*n* = 99)	4 (4.0%)	1.04 (0.28–2.65)	0.80 (0.27–2.39)	0.69	
ILR—systolic B*p* < 150 (*n* = 202)	9 (4.5%)	1.15 (0.53–2.19)	1		
ILR—systolic BP ≥ 150 (*n* = 239)	12 (5.0%)	1.3 (0.67–2.28)	1.13 (0.48–2.68)	0.44	
Control—creatinine < 100 µM (*n* = 433)	21 (4.8%)	2.08 (1.29–3.18)	1		
Control—creatinine ≥ 100 µM (*n* = 143)	5 (3.5%)	1.48 (0.48–3.44)	0.71 (0.27–1.88)	0.49	
Control—systolic B*p* < 150 (*n* = 286)	10 (3.5%)	1.5 (0.72–2.77)	1		
Control—systolic BP ≥ 150 (*n* = 292)	16 (5.5%)	2.33 (1.33–3.78)	1.53 (0.69–3.36)	0.40	
Antiplatelets					
All (*n* = 3,374)	139 (4.1%)				
All—creatinine < 100 µM (*n* = 2,536)	101 (4.0%)	0.83 (0.67–1.01)	1		
All—creatinine≥100 µM (*n* = 824)	38 (4.6%)	0.96 (0.68–1.32)	1.16 (0.80–1.69)	0.43	0.49
All—systolic B*p* < 150 (*n* = 1,747)	71 (4.1%)	0.84 (0.66–1.06)	1		
All—systolic BP≥150 (*n* = 1624)	68 (4.2%)	0.88 (0.68–1.11)	1.04 (0.75–1.45)	0.81	0.11
ILR—creatinine < 100 µM (*n* = 598)	30 (5.0%)	1.05 (0.71–1.49)	1		
ILR—creatinine≥100 µM (*n* = 204)	14 (6.9%)	1.45 (0.79–2.44)	1.39 (0.74–2.63)	0.31	
ILR—cystolic B*p* < 150 (*n* = 406)	18 (4.4%)	0.92 (0.55–1.46)	1		
ILR—cystolic BP ≥ 150 (*n* = 399)	26 (6.5%)	1.37 (0.89–2.00)	1.47 (0.81–2.68)	0.21	
Control—creatinine < 100 µM (*n* = 1,938)	71 (3.7%)	0.76 (0.59–0.96)	1		
Control—creatinine ≥ 100 µM (*n* = 620)	24 (3.9%)	0.80 (0.51–1.19)	1.06 (0.66–1.68)	0.82	
Control—systolic B*p* < 150 (*n* = 1341)	53 (4.0%)	0.82 (0.61–1.07)	1		
Control—systolic BP ≥ 150 (*n* = 1225)	42 (3.4%)	0.72 (0.52–0.97)	0.88 (0.59–1.32)	0.53	

Abbreviations: BP, blood pressure; CI, confidence interval; HR, hazard ratio; ILR, implantable loop recorder; OAC, oral anticoagulants.
[a]Interaction between the exposure and randomization group
Note: All participants were included in the analysis and could appear in several exposure groups, pending change in medical treatment. In each exposure group, rates were calculated as time from baseline or start of the treatment until event or censoring which included initiation of treatment for the "no treatment"-exposure group.

Supplementary Table S3 Sensitivity analysis: Risk of major bleeding after initiation of incident antiplatelets

Antiplatelets	Unadjusted HR (95% CI)	*p*	Adjusted HR[a] (95% CI)	*p*
All	1.37 (1.04–1.80)	0.024	1.14 (0.85–1.54)	0.38
Control	1.20 (0.87–1.65)	0.27	1.03 (0.73–1.47)	0.86
ILR	1.94 (1.15–3.27)	0.012	1.45 (0.83–2.55)	0.20

Abbreviations: CI, confidence interval; HR, hazard ratio; ILR, implantable loop recorder; SAE, systemic arterial embolism; SBP, systolic blood pressure.
[a]Adjusted for age, sex, SBP, stroke or TIA or SAE, creatinine, and alcohol consumption; TIA, transient ischemic attack.

 © 2024. The Author(s).
